# Usefulness of Computed Tomography Image Processing by OsiriX Software in Detecting Wooden and Bamboo Foreign Bodies

**DOI:** 10.1155/2017/3104018

**Published:** 2017-10-03

**Authors:** Shinpei Matsuda, Hitoshi Yoshimura, Hisato Yoshida, Takashi Ryoke, Takashi Yoshida, Naoyuki Aikawa, Kazuo Sano

**Affiliations:** ^1^Department of Dentistry and Oral Surgery, Unit of Sensory and Locomotor Medicine, Division of Medicine, Faculty of Medical Sciences, University of Fukui, Fukui, Japan; ^2^Department of Applied Electronics, Faculty of Industrial Science and Technology, Tokyo University of Science, Tokyo, Japan

## Abstract

**Objective:**

The aim of this study was to evaluate the usefulness of reconstructed computed tomography (CT) images using OsiriX software in detecting wooden and bamboo foreign bodies.

**Methods:**

Four sizes of wet and dry wooden and bamboo foreign bodies were selected to be analyzed. Those in the air and in the head of edible swine were scanned with a multidetector row CT scanner. The images were evaluated with OsiriX software in the bone and the abdomen window setting as unprocessed images. Three-dimensional rendered images assigned colors and opacity by a 16-bit color look-up table (CLUT) editor in OsiriX software were evaluated as processed images.

**Results:**

In the unprocessed images, dry and wet foreign bodies in the air were not detected except a part of wet wooden foreign bodies, and all the dry and wet foreign bodies in the swine's head mimicked air with linear shapes. In the processed images, all the dry and wet foreign bodies in the air were detected clearly, and all the wooden and some of the bamboo foreign bodies in the swine's head were detected clearly.

**Conclusions:**

CT images processed using OsiriX software, especially with a CLUT editor, were useful in detecting wooden and bamboo foreign bodies.

## 1. Introduction

Foreign bodies retained in the body often present a diagnostic challenge for clinicians. Overall, 38% of patients with foreign bodies were misdiagnosed by the initial treating physician [1]. Diagnosis of wooden foreign bodies (WFBs) is more difficult than other kinds of foreign bodies because detection by imaging examination is difficult despite advances in techniques [2]. Further, the detection of bamboo foreign bodies (BFBs) is as difficult as that of WFBs [3]. WFBs and BFBs provide good media for microorganisms [3]. Thus, those remaining in the body usually cause infection and may result in cellulitis, abscess, and fistula formation [2]. Organic foreign body-related infection may cause serious and potentially lethal consequences in cases of penetration into the oral and maxillofacial region. Therefore, accurate diagnosis is required at the first visit to a physician.

Computed tomography (CT) is often performed initially in the emergency department to detect foreign bodies [4]. However, under the standard window and level setting on CT, WFBs mimic air because the interstices of the dry wood are predominantly filled with air, leading to misdiagnosis [2, 4]. Moreover, there is a possibility that BFBs can be missed on CT because they are isodense with fat [5]. Therefore, it is essential to establish a method for detecting WFBs and BFBs clearly using CT images.

OsiriX software is image-processing software dedicated to Digital Imaging and Communications in Medicine (DICOM) images. It supports various medical imaging examinations such as CT, magnetic resonance imaging (MRI), positron emission tomography, and ultrasonography [6]. Although OsiriX can provide useful information clinically, there have been no reports of its utility in detecting foreign bodies.

The aim of this study was to evaluate the usefulness of reconstructed CT images using OsiriX software in detecting WFBs or BFBs.

## 2. Materials and Methods

WFBs and BFBs were embedded in the head of edible swine slaughtered on the day of the experiment. Foreign bodies in the air and in the swine's head were scanned by a multidetector row CT scanner. CT image data were converted into the DICOM format. Processed images and unprocessed images were evaluated using OsiriX software.

### 2.1. Foreign Bodies

#### 2.1.1. Dry Wooden and Bamboo Foreign Bodies

The authors used 4 sizes of Japanese cypress as dry WFBs: (1) a cylindrical shape 10 mm in diameter and 70 mm in length; (2) a square pillar shape 10 mm in width, 10 mm in height, and 70 mm in length; (3) a square pillar shape 5 mm in width, 5 mm in height, and 70 mm in length; and (4) a square pillar shape 3 mm in width, 3 mm in height, and 70 mm in length. The authors also used 4 sizes of bamboo as dry BFBs: (5) a cylindrical shape 5 mm in diameter and 70 mm in length; (6) a square pillar shape 5 mm in width, 2 mm in height, and 70 mm in length; (7) a cylindrical shape 3 mm in diameter and 70 mm in length; and (8) a cylindrical shape 1.8 mm in diameter and 70 mm in length.

#### 2.1.2. Wet Wooden and Bamboo Foreign Bodies

The WFBs described above were immersed in water for an hour and used as wet WFBs: (9) a cylindrical shape 10 mm in diameter and 70 mm in length; (10) a square pillar shape 10 mm in width, 10 mm in height, and 70 mm in length; (11) a square pillar shape 5 mm in width, 5 mm in height, and 70 mm in length; and (12) a square pillar shape 3 mm in width, 3 mm in height, and 70 mm in length. The BFBs described above were immersed in water for an hour and used as wet BFBs: (13) a cylindrical shape 5 mm in diameter and 70 mm in length; (14) a square pillar shape 5 mm in width, 2 mm in height, and 70 mm in length; (15) a cylindrical shape 3 mm in diameter and 70 mm in length; and (16) a cylindrical shape 1.8 mm in diameter and 70 mm in length.

#### 2.1.3. CT Scan

An 8-row multidetector CT scanner (ECLOS-8S; Hitachi Medico, Tokyo, Japan) was used. The CT scanning parameters were as follows: tube voltage 120 kV, tube current 150 mA, slice thickness 0.625 mm, collimation thickness 0.625 × 8 mm, pitch 1.125, and field of view 300 mm.

### 2.2. Imaging Analysis and Image Processing Using OsiriX

The unprocessed images were evaluated using OsiriX software in the bone window setting (window width: 1500 HU; window level: 300 HU) and the abdomen window setting (window width: 350 HU; window level: 40 HU). The processed DICOM images were rendered as three-dimensional images and were assigned colors and opacity by a 16-bit color look-up table (CLUT) editor to identify WFBs and BFBs from air and swine tissue [6].

The visibility was evaluated in 5 grades based on previous literature [7, 8] as follows: (+4) excellent resolution of details, excellent visibility, and good demarcation from surroundings; (+3) good resolution of details, demarcation from surroundings, and clear visibility; (+2) insufficient resolution of details, unsatisfactory visibility, and inadequate demarcation; (+1) details not resolved, bad demarcation from surroundings, and poor visibility, and (0) invisible. Three oral and maxillofacial surgeons evaluated the quality of images, and the average of the results was recorded.

## 3. Results

### 3.1. Unprocessed Images

#### 3.1.1. Dry Wooden and Bamboo Foreign Bodies in the Air (Figure 1)

All the dry WFBs and BFBs were not detected at the bone and the abdomen window setting in OsiriX.

#### 3.1.2. Wet Wooden and Bamboo Foreign Bodies in the Air (Figure 2)

Only a part of numbers 9, 10, and 11 was visible, and the other wet WFBs and BFBs were not detected at the bone and the abdomen window setting in OsiriX.

#### 3.1.3. Dry Wooden and Bamboo Foreign Bodies in the Swine's Head (Figures 3 and 4)

All the dry WFBs and BFBs mimicked air, with a linear shape at the bone and the abdomen window setting in OsiriX.

#### 3.1.4. Wet Wooden and Bamboo Foreign Bodies in the Swine's Head

All the wet WFBs and BFBs mimicked air, with a linear shape at the bone and the abdomen window setting in OsiriX.

### 3.2. Processed Images Using OsiriX

#### 3.2.1. Dry Wooden and Bamboo Foreign Bodies in the Air (Figure 1, Table 1)

All the dry WFBs and BFBs were detected clearly because of changes in color and opacity using the OsiriX 16-bit CLUT editor.

#### 3.2.2. Wet Wooden and Bamboo Foreign Bodies in the Air (Figure 2, Table 2)

All the wet WFBs and BFBs were detected clearly because of changes in color and opacity using the OsiriX 16-bit CLUT editor.

#### 3.2.3. Dry Wooden and Bamboo Foreign Bodies in the Swine's Head (Figures 3 and 4, Table 1)

All the dry WFBs and BFBs with a cylindrical shape, diameter of 5 mm, and length of 70 mm were detected, with clear boundaries between WFBs and the swine's soft tissues because of changes in the color and opacity using the OsiriX 16-bit CLUT editor.

#### 3.2.4. Wet Wooden and Bamboo Foreign Bodies in the Swine's Head (Table 2)

All the wet WFBs and BFB with a cylindrical shape, diameter of 5 mm, and length of 70 mm were detected with clear boundaries between WFB and the swine's soft tissues because of changes in color and opacity using the OsiriX 16-bit CLUT editor.

## 4. Discussion

Detection of WFBs or BFBs by imaging examinations is more difficult than that of other foreign bodies such as metal, stone, and graphite [3, 7, 8]. Anderson et al. reported that only 15% of WFBs were visualized on plain X-ray examinations [1]. In this study, while dry WFBs and BFBs in the air could not be identified in the bone or the abdomen window setting except for a part of the wet foreign body, processing CT images using OsiriX software enabled visualizing them. These results suggested that postprocessing images may be useful for detecting WFBs or BFBs in air-filled spaces such as the nasal cavity, maxillary sinus, and pharynx. Only a part of numbers 9, 10, and 11 in the air was visible in the unprocessed images. The authors considered that this result seems to be related to the fact that the stump of wood was superior in water absorbency.

WFBs or BFBs in the swine's head mimicked air with a linear shape in the unprocessed images. In the postprocessing images, regardless of whether they were dry or wet, all the WFBs and one BFB in the swine's head were identified and distinguished from the swine tissues. When foreign bodies penetrate the soft tissues, air may also be embedded at the same time. Therefore, it is important to distinguish retained WFBs or BFBs from the air to avoid misdiagnosis. The results of this study suggested that processed images using OsiriX software are more useful than CT images in the bone or the abdomen window settings in detecting WFBs or BFBs embedded in the soft tissues. The visibility of BFBs depended on their thickness. Three sizes of BFBs that were shorter than 2 mm or with a diameter of 3 mm or less could not be confirmed. In this study, the shapes of WFBs and BFBs were different because commercially available wood and bamboo materials were used as samples of uniform shape. Therefore, the difference in visibility between WFBs and BFBs could not be evaluated. In the future, it is necessary to perform experiments using the same size of the foreign bodies or under varying imaging conditions.

OsiriX software is a DICOM viewer program for Apple Macintosh, and it is designed for the navigation and visualization of multimodality and multidimensional images [9]. It is an open-source program available from the Internet. Therefore, it is used by researchers and clinicians worldwide. The three-dimensional OsiriX viewer offers rendering modes as follows: multiplanar reconstruction, surface rendering, volume rendering, and maximum intensity projection [9]. OsiriX software can display a histogram of target images, showing the density value scale on the horizontal axis and the opacity on the vertical axis [6]. OsiriX's 16-bit CLUT editor is a software tool for manipulating thresholding colors [10]. A colored curve is displayed in the histogram of the 16-bit CLUT editor, and the color of the whole curve or of some specific points and the opacity can be changed [6]. It can improve the visibility and facilitate the analysis of a region of interest. Clinically, OsiriX's usefulness in pre- or postoperative evaluation has been reported in previous literature [10, 11]. The application range of OsiriX, including the detection of foreign bodies, will further expand in the future.

The selection of an imaging modality to detect organic foreign bodies remains controversial. Javadrashid et al. reported that WFBs could only be detected using ultrasonography [8]. Mohammadi et al. found that ultrasonography was useful for the detection and localization of radiolucent foreign bodies in soft tissue [12]. Ultrasonography is noninvasive, and images are obtained in real time. However, images cannot be obtained when there is bone or air. Furthermore, in ultrasonography, a special probe is necessary depending on the examination site, and the examiner's skills are required to detect small foreign bodies. Clinical evaluation may fail to elicit a history of penetrating trauma and may lead to misdiagnosis because patients often visit the hospital for evaluation several months or years after the initial injury [2]. CT and MRI are more suitable than ultrasonography because they are necessary to confirm the cause of clinical symptoms in various examination sites in such cases. Dalley reported that MRI may be more sensitive to detect dry WFBs [13]. However, MRI is inferior to CT for detecting various foreign bodies, and it is dangerous if there is a possibility of the presence of metallic foreign bodies [2, 8, 14]. Ingraham et al. reported that MRI could not be a first-choice modality to detect foreign bodies because it is more costly and time-consuming than other imaging examinations, and CT is useful for localizing foreign bodies and determining their relationship to the surrounding structures [14]. Additionally, CT is superior to other examinations in the detection of trauma, such as the bone fracture, which may simultaneously accompany the embedding of foreign bodies. Thus, we consider that CT is the most useful modality for detecting foreign bodies. However, WFBs and BFBs mimic air at CT in conventional bone or soft tissue window setting, and the shapes of those areas have been the key to diagnosis [2, 4, 5]. OsiriX can be assigned colors and opacity to WFBs and BFBs to distinguish them from air and swine tissue. We considered that clinicians can accurately diagnose retaining WFBs or BFBs without relying on shape or slight changes in attenuation using this study's method.

In this study, we used the head of an edible swine slaughtered on the day of the experiment to perform the experiment with conditions close to a living body. However, the experimental models lacked the ability to reproduce inflammatory reactions around foreign bodies [8]. Swelling in living bodies may occur due to embedded foreign bodies. It may interfere with imaging; however, it may suggest the presence of remaining foreign bodies [8]. Additionally, there is an anatomical difference between humans and swine. Thus, verification of the usefulness of this method in human clinical cases is necessary in the future.

## 5. Conclusions

Regardless of whether the foreign bodies are dry or wet, CT images processed using OsiriX software, especially with the CLUT editor, are useful in detecting WFBs or BFBs. In this method, the visibility of WFBs or BFBs in soft tissue depends on their thickness.

## Figures and Tables

**Figure 1:**
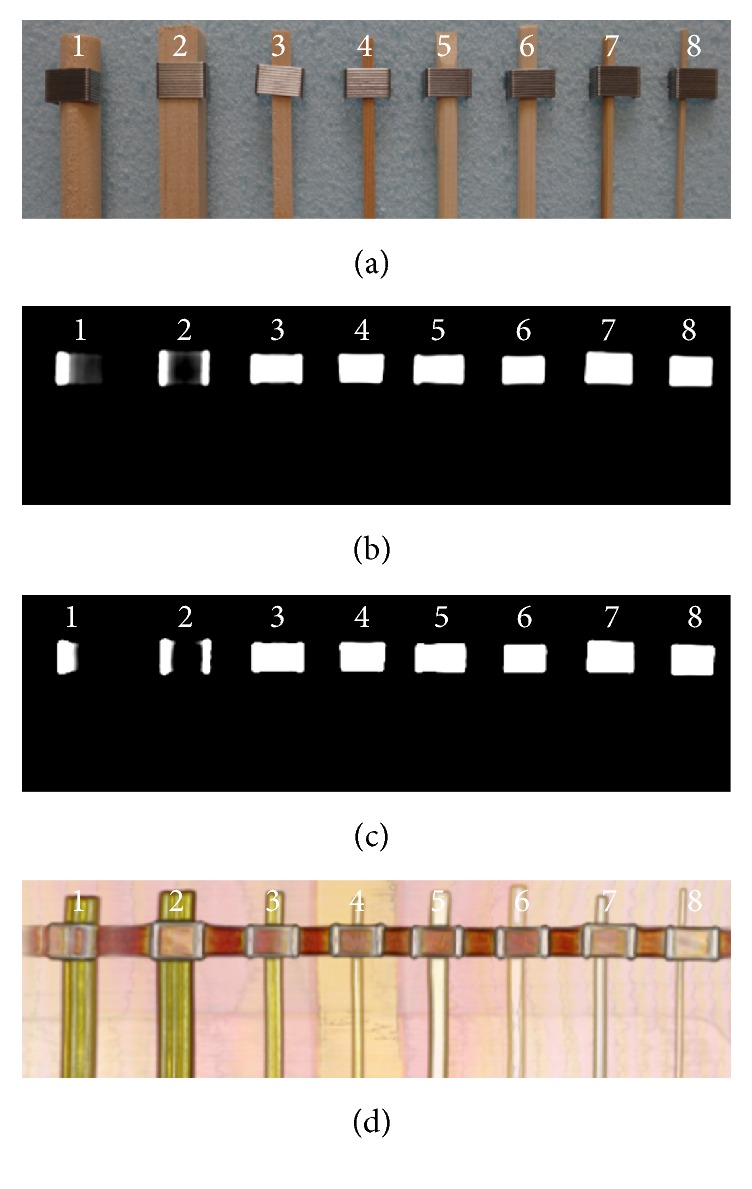
Dry foreign bodies in air. (a) Foreign bodies were placed in a sequence on Styrofoam from 1 to 8 from the left. The upper parts of the foreign bodies were fixed with metal. (b) The bone window setting. (c) The abdomen window setting. (d) The processed image using OsiriX software.

**Figure 2:**
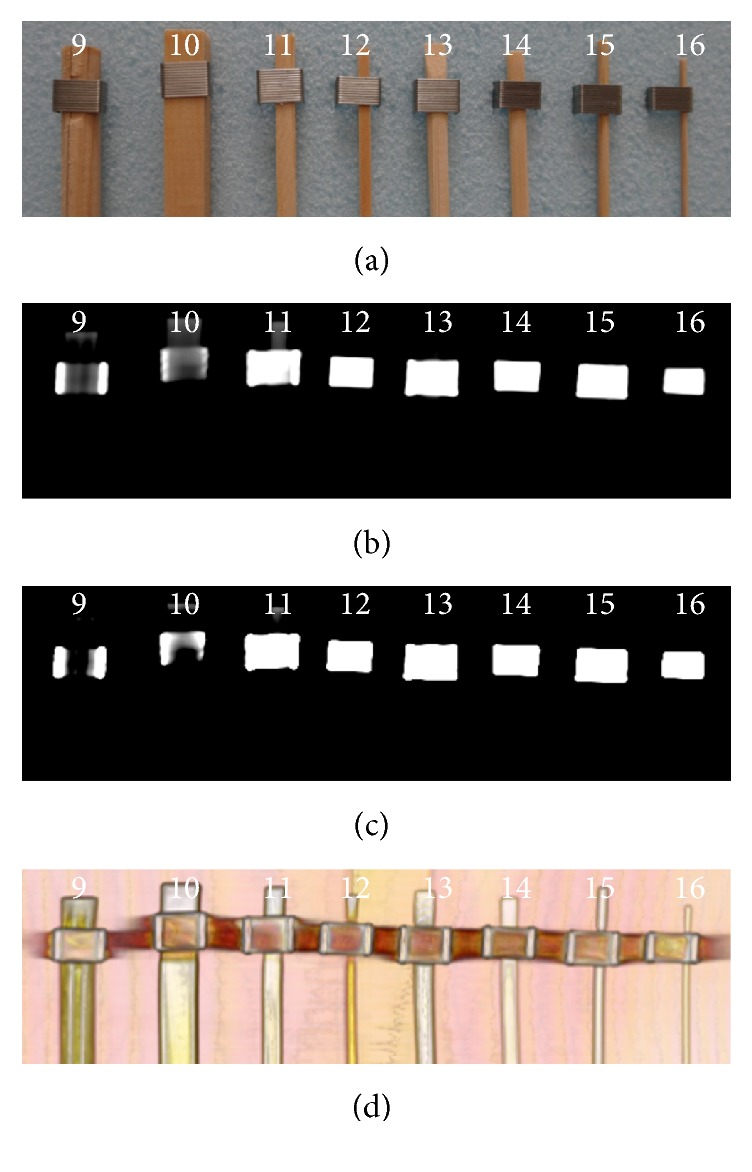
Wet foreign bodies in air. (a) Wet foreign bodies were placed in a sequence on Styrofoam from 9 to 16 from the left. The upper parts of the foreign bodies were fixed with metal. (b) The bone window setting. (c) The abdomen window setting. (d) The processed image using OsiriX software.

**Figure 3:**
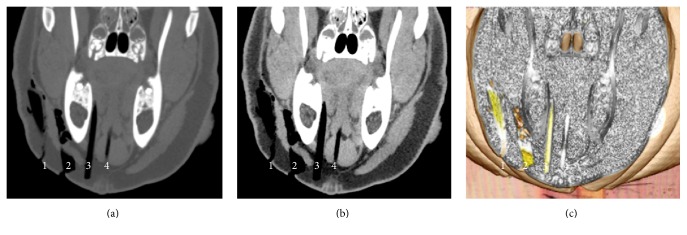
Dry WFB embedded in the swine's head. (a) The bone window setting. (b) The abdomen window setting. (c) The processed image using OsiriX software.

**Figure 4:**
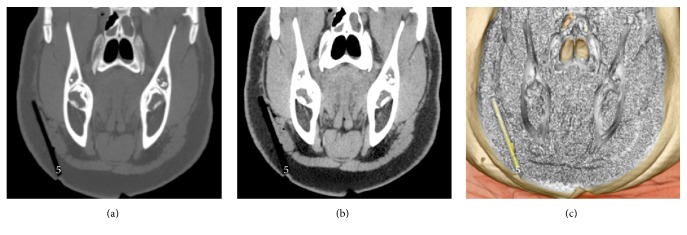
Dry BFB embedded in the swine's head. This figure shows number 5 foreign body. (a) The bone window setting. (b) The abdomen window setting. (c) The processed image using OsiriX software.

**Table 1:** The visibility of processed images of wooden and bamboo foreign bodies.

Dry foreign bodies	Visibility in air	Visibility in swine's head
WFB		
1	+4	+4
2	+4	+4
3	+4	+4
4	+4	+4
BFB		
5	+4	+4
6	+4	+1
7	+4	0
8	+4	0

**Table 2:** The visibility of processed images of wooden and bamboo foreign bodies.

Wet foreign bodies	Visibility in air	Visibility in swine's head
WFB		
9	+4	+4
10	+4	+4
11	+4	+4
12	+4	+4
BFB		
13	+4	+4
14	+4	+1
15	+4	0
16	+4	0
